# Demographics, treatment patterns, and healthcare resource utilization in Parkinson's disease: a real-world data study using a claims database

**DOI:** 10.1055/s-0045-1814375

**Published:** 2026-01-25

**Authors:** Alexandre Battaglini Chehin, Luciana Rahal Abrahão, Marcos Augusto Mira Fuga, Ana Beatriz Machado de Almeida, Angelica Carreira dos Santos, Jacy Bezerra Parmera

**Affiliations:** 1 AbbVie Inc, North Chicago IL, United States of America.; 2 IQVIA Brasil, São Paulo SP, Brazil.; 3 Universidade de São Paulo, Faculdade de Medicina, Hospital das Clínicas, Departamento de Neurologia, São Paulo SP, Brazil.; 4 Hospital Israelita Albert Einstein, São Paulo SP, Brazil.

**Keywords:** Parkinson Disease, Unified Health System, Health Resources, Therapeutics

## Abstract

**Background:**

Parkinson's disease (PD) is a progressive neurodegenerative disorder associated with substantial disability, morbidity, and mortality. Timely diagnosis and treatment are essential to mitigate its impact. Despite its burden, real-world data on PD in Brazil remain limited.

**Objective:**

To describe the demographic and clinical profile of individuals with PD treated in the Brazilian public healthcare system and to evaluate patterns of treatment and healthcare resource utilization (HCRU).

**Methods:**

The present observational, retrospective, longitudinal study analyzed data from the Brazilian public healthcare system (Sistema Único de Saúde, SUS, in Portuguese) between January 2013 and December 2022. Patients aged ≥ 20 years with at least 2 core procedures coded for PD (ICD-10 G20) were included. Inpatient and outpatient datasets were analyzed separately.

**Results:**

A total of 53,674 PD patients were identified. The mean age at diagnosis was 65.4 years, with a slight male predominance (53.0%). Most patients (47.0%) had more than 6 outpatient visits, and 44.4% had 1 or 2 hospitalizations. The most frequent procedures were PD treatment (inpatient) and physical therapy (outpatient). The most used medications were pramipexole (45.6%), amantadine (26.0%), and entacapone (17.1%).

**Conclusion:**

The present study provides valuable insights into the demographic and clinical profile of PD patients in Brazil, highlighting frequent procedures and treatment patterns. A key limitation is the non-capture of basic PD medications, such as levodopa, which are often dispensed outside the analyzed datasets. These findings underscore the need for improved data integration and access to comprehensive PD care within the public health system.

## INTRODUCTION

Parkinson's disease (PD) is a chronic, progressive neurodegenerative disorder that affects ∼ 1 to 3% of individuals over the age of 65. It is clinically characterized by motor symptoms such as bradykinesia, resting tremor, rigidity, and postural instability, as well as a range of non-motor manifestations that contribute to significant morbidity and reduced quality of life. 1 2 Among neurological disorders, PD has shown one of the fastest-growing prevalence rates globally, contributing to substantial healthcare burden and increased mortality. 3 4

In Brazil, the prevalence of PD is rising, largely attributed to the aging population. 5 As the disease advances, patients often experience fluctuations in motor response and the emergence of motor and non-motor complications that are less responsive to conventional pharmacological therapies, including monoamine oxidase B (MAO-B) inhibitors, dopamine agonists, and catechol-O-methyltransferase (COMT) inhibitors. 6 7 8 9 This clinical stage is commonly referred to as advanced PD (aPD).

Despite progress in neuroimaging and molecular research, PD diagnosis remains primarily clinical, as no definitive biomarker or imaging modality has been established for routine use. 10 Techniques such as magnetic resonance imaging (MRI), positron-emission tomography (PET), and dopamine transporter (DAT) scans may support diagnosis in selected cases. 11 12 13 However, in public healthcare settings, particularly those with limited resources, access to multidisciplinary care is often constrained, leading to delays in diagnosis and suboptimal disease management. 14

Although PD is incurable, a range of therapeutic strategies—including pharmacological and surgical interventions—are available to manage symptoms and improve patient outcomes. 15 Nevertheless, there is a paucity of real-world data on the clinical characteristics, treatment patterns, and healthcare resource utilization (HCRU) of PD patients within the Brazilian Unified Health System (Sistema Único de Saúde, SUS, in Portuguese).

The current study aims to describe the demographic and clinical profile of individuals with PD treated in SUS and to evaluate patterns of treatment and HCRU, thereby contributing to a better understanding of disease management in the Brazilian context.

## METHODS

The present is an observational retrospective longitudinal study in SUS. Cases were identified based on the administrative claims databases from the Computer Science Department of the SUS (Departamento de Informática do SUS, DATASUS, in Portuguese) that contain information and statistics from all municipalities in Brazil and are publicly available.

To improve diagnostic specificity and minimize misclassification, only patients aged ≥ 20 years with at least 2 recorded core procedures related to PD ( *International Classification of Diseases, Tenth Revision* [ICD-10], code G20) were included ( **Supplementary Material** , available at https://www.arquivosdeneuropsiquiatria.org/wp-content/uploads/2025/10/ANP-2025.0042-Supplementary-Material.docx ). This criterion, commonly used in administrative database studies, helps ensure a higher likelihood of confirmed diagnosis by reducing the inclusion of isolated or miscoded cases.

To exclude secondary parkinsonism, only ICD-10 code G20 was used, and codes for other parkinsonian syndromes (such as G21–G26) were not included. Patients were identified between January 2013 and December 2022 and followed up from their first PD-related claim until loss to follow-up or the end of the study period, whichever occurred first.

The Hospital Information System (Sistema de Informações Hospitalares, SIH, in Portuguese) and Ambulatory Information System (Sistema de Informações Ambulatoriais, SIA, in Portuguese ), which provide inpatient and outpatient data, respectively, are separate datasets and were linked at the patient level using a probabilistic linkage approach. The linkage used in this analysis was developed by Techtrials Pesquisa e Tecnologia Ltda. and used different combinations of patient information from both databases, such as date of birth, sex, and ZIP code, identifying patients in both systems. This method relies on multiple steps with different combinations of patient information from both databases, making it possible to identify or link patient data in both systems while maintaining the de-identified nature of the database.

Comorbidities were assessed using ICD-10 codes recorded in both inpatient (SIH) and outpatient (SIA) datasets over the entire study period (2013–2022). Patients were classified as having comorbidities if any relevant ICD-10 codes were present in their records during this timeframe.

The outcomes were summarized by calendar year or by year after the index date using descriptive statistics. Continuous variables were described using measures of central tendency (mean, median) and measures of spread, including range, and quartiles. Categorical variables were described as counts and percentages. The treatment patterns were assessed by the number and proportion of the different drugs used to treat PD considering only the Outpatient Procedure Authorization (Autorização de Procedimentos Ambulatoriais, APAC, in Portuguese) claims. As such, this dataset does not include medications provided through the Basic Component of Pharmaceutical Assistance, such as levodopa-benserazide and levodopa-carbidopa. Consequently, the analysis of pharmacological treatment patterns in the present study reflects only a subset of patients receiving advanced therapies and does not capture the full spectrum of PD pharmacological management within SUS.

Missing data was reported, and no data imputation was performed. All computations and generation of tables, listings, graphics, and data for figures were performed using the Python (Python Software Foundation) software, version 3.11.0.

## RESULTS

### Study population

**Figure 1:**
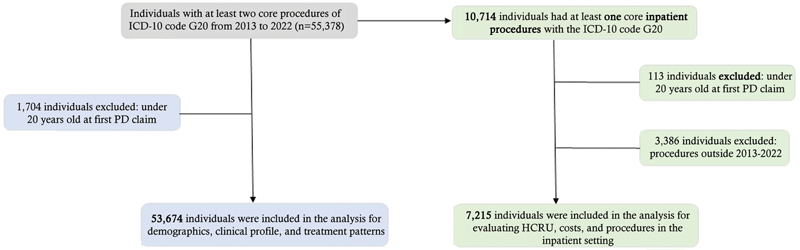
Flowchart of the inclusion and exclusion criteria of the present study.

A total of 55,378 individuals with at least 2 core procedures of ICD-10 code G20 were identified in the database from 2013 to 2022. Of these, 1,704 (3.1%) were excluded because they were under 20 years old at the 1st PD claim. Thus, the final cohort of this study consisted of 53,674 patients. From the entire cohort, 10,728 patients had at least 2 core inpatient procedures with the ICD-10 code G20. Out of these, 3,513 were excluded because they were under 20 years old at the time of the 1st PD claim. Therefore, the final cohort for evaluating HCRU, and procedures in the inpatient setting consisted of 7,215 patients ( Figure 1 ).

### Demographics and clinical profile

Among the identified PD patients ( *n* = 53,674), the mean age at the index date was of 65.4 ± 13.6 years. Regarding gender, there was a slight male predominance (53.0%). Most of the population resided in the Southeast region of Brazil (48.8%) and did not have comorbidities (61.3%) ( Table 1 ).

**Table 1:** Demographic profile of patients with Parkinson's disease (PD)

		PD patients (N = 53,674)
**Age at index date (years)**	*n valid*	**53,674**
	Mean	65.4 ± 13.6
	Median (Q1; Q3)	67 (57; 75)
	Minimum	20
	Maximum	108
**Age group** – N (%)	*n valid*	**53,674**
	20–29 years	863 (1.6)
	30–39 years	1,630 (3.0)
	40–49 years	4,094 (7.6)
	50–54 years	3,956 (7.4)
	55–59 years	5,422 (10.1)
	60–64 years	7,039 (13.1)
	65–69 years	7,950 (14.8)
	70–74 years	8,204 (15.3)
	75–79 years	7,136 (13.3)
	≥ 80 years	7,380 (13.7)
**Gender** – N (%)	*n valid*	**53,674**
	Male	28,443 (53.0)
	Female	25,053 (46.7)
	Missing	178 (0.3)
**Region of residence** – N (%)	*n valid*	**53,674**
	South	6,229 (11.6)
	Southeast	26,189 (48.8)
	North	1,878 (3.5)
	Northeast	14,771 (27.5)
	Midwest	4,190 (7.8)
	Missing	417 (0.8)
**Comorbidities** – N (%)	*n valid*	**53,674**
	No	32,903 (61.3)
	Yes	20,771 (38.7)
**Follow-up time (years)**	Mean	1.8 ± 2.3
	Median (Q1; Q3)	0.66 (0.1;2.8)
	Minimum	0.005
	Maximum	9.79

### Healthcare Resource Utilization (HCRU)

From the entire cohort, 53,075 patients had at least 1 outpatient visit due to PD, with a total of 835,235 visits during the study period. Most of the patients (47.0%) had more than 6 outpatient visits, with a mean number of outpatient visits related to PD of 15.8 (60.9) ( Table 2 ).

**Table 2:** Healthcare resource utilization of patients with Parkinson's disease in the Brazilian Unified Health System

***Outpatient setting***	
**Patients with at least one outpatient visit** – N (%)	**53,075 (100)**
**Outpatient visits, per patient (n)**	
Mean	15.8 ± 60.9
Median (IQR)	6 (12)
Median (95%CI) PPPY	27.10 [26.8;27.4]
Patients with 1–2 outpatient visits	12,156 (22.9%)
Patients with 3–6 outpatient visits	15,975 (30.1%)
Patients with > 6 outpatient visits	24,944 (47.0%)
**Total number of outpatient visits (n)**	**835,235**
***Inpatient setting***	
Patients with at least one hospitalization – N (%)	**7,215 (100%)**
Hospital admissions, per patient (n)	
Mean	5.1 ± 11.3
Median (IQR)	3 (4)
Median (95% CI) PPPY	18.8 [18.3;19.4]
Patients with 1–2 hospitalizations	3,207 (44.4%)
Patients with 3–6 hospitalizations	2,600 (36.0%)
Patients with > 6 hospitalizations	1,408 (19.5%)
Total number of hospitalization (n)	9,101
Total length of stay (days)	
Mean	12 ± 59
Median (IQR)	4 (7)

Abbreviations: IQR, interquartile range; PPPY, per patient per year.

In the inpatient setting, 7,215 patients had at least 1 hospitalization due to PD, and there was a total of 9,101 hospitalizations during the study period. Most patients (44.4%) had between 1 and 2 hospitalizations, with the average (SD) number of hospital admissions related to PD being 5.10 ± 11.3 ( Table 2 ).

It is notable that in 2020, the number of procedures performed decreased by 30.1% and 25.8% in the inpatient and outpatient settings, respectively. In the inpatient setting, the most common procedures performed on PD patients were PD treatment, pharmacological adjustment of acute neurological situations, and treatment of patients under long-term care for neurological disease. Parkinson's disease treatment was performed on most patients (4,501; 62.4%), with an average of 467 patients per year ( Table 3 ).

**Table 3:** Procedures performed for patients with Parkinson's disease in the Brazilian Unified Health System by calendar year

	Total	2013	2014	2015	2016	2017	2018	2019	2020	2021	2022
***Inpatient setting***											
Parkinson's disease treatment	**4,501**	553	700	594	547	469	418	420	319	323	329
Treatment of patient under long-term care for neurological disease	**455**	28	45	50	40	47	59	60	48	70	37
Sequential procedures in neurosurgery	**374**	–	7	19	45	59	70	79	39	47	42
Pharmacological adjustment of acute neurological situations	**718**	55	61	67	145	89	64	73	56	69	47
Treatment with multiple surgeries	**281**	15	39	30	37	33	37	33	35	30	13
Brain implant for pacing electrode	**450**	50	73	63	63	39	43	54	28	38	39
Diagnostic and/or emergency call in medical clinic	**396**	32	50	45	55	58	36	44	24	21	34
Implant of pulse generator for brain stimulation (including connector)	**265**	10	27	23	40	24	34	44	30	38	42
Exchange of pulse generator for brain stimulation	**88**	–	–	3	–	10	14	18	8	24	17
Home care	**43**	–	–	17	15	9	11	3	1	–	2
Neurodegenerative diseases treatment	**19**	–	–	1	–	–	4	5	4	3	2
Dental treatment for patients with special needs	**12**	5	1	4	2	–	–	–	–	–	–
Treatment of central motor neurons disease with or without amyotrophy	**14**	–	–	2	6	5	1	–	–	–	–
Rehabilitation treatment	**6**	–	–	–	–	1	2	1	–	–	2
Treatment of complications of surgical or clinical procedures	**17**	–	–	–	1	3	1	6	2	3	1
Treatment of abnormal movements via stereotactic surgery with microrecord	**7**	–	–	1	1	–	3	1	1	–	–
Conservative treatment of central or neoplastic pain	**3**	–	–	–	–	–	–	1	1	–	1
Treatment of abnormal movements via stereotactic surgery	**7**	–	–	3	–	–	1	2	–	–	1
Treatment of not controlled epileptic crisis	**6**	–	–	–	1	2	1	1	–	–	1
Hypertensive crisis treatment	**5**	–	–	1	1	2	–	–	–	1	–
Others	**232**	10	21	24	21	22	31	34	18	30	25
***Outpatient setting***											
Physical therapy in patient with neurokinetic functional disorders without systemic complications	**17,616**	1,853	1,992	2,583	2,864	3,107	3,355	3,349	2,496	2,852	3,008
Physical therapy in patient with motor changes	**16,130**	1,487	1,698	2,152	2,290	2,318	2,665	2,886	2,066	2,815	3,138
Physical therapy in patient pre- and postsurgery with skeletal muscle dysfunction	**10,899**	1,070	1,507	2,175	1,976	1,630	1,789	1,789	1,197	1,427	1,321
Physical therapy in patient with neurokinetic functional disorders with systemic complications	**11,903**	1,141	1,293	1,638	1,880	2,191	2,317	2,376	1,610	1,803	2,099
Patient intensive treatment in physical rehabilitation (1 patient per day shift; 20 calls per month)	**5,253**	523	623	908	954	1,034	1,173	1,284	1,014	1,134	1,108
Intensive care - follow up of patient in physical rehabilitation (1 patient per day shift; 15 calls per month)	**2,150**	243	320	278	273	350	472	547	447	458	442
Consultation of higher-level professional in primary care (except medical)	**116**	–	–	1	7	27	23	7	43	7	12
Medical consultation on specialized attention	**3,605**	–	74	184	387	521	755	1,158	1,051	1,449	1,396
Computed tomography of skull	**1,231**	130	116	134	162	181	148	139	105	118	116
Magnetic resonance of skull	**2,490**	158	193	261	301	329	295	350	209	313	287
Care: monitoring on rehabilitation in multiple deficiencies	**1,462**	9	24	56	56	114	143	177	123	612	572
Physical therapy in patient pre- postneurosurgery	**825**	97	92	119	167	183	138	110	55	95	99
Teleconsultation by higher level professionals in specialized attention (except medical)	**980**	–	–	–	–	–	–	–	–	716	407
Household assistance by multi professional team in specialized attention	**332**	52	60	58	48	64	65	50	38	55	52
Individual therapy	**440**	–	14	28	18	43	59	69	56	181	146
Household assistance by multi professional team	**189**	53	41	47	50	26	19	25	4	10	22
Functional muscle evaluation	**-**	–	–	–	–	–	–	–	–	–	–
Physical therapy in patient with neuromotor development disorders	**-**	–	–	–	–	–	–	–	–	–	–
Medical care in emergency care unit	**133**	–	–	–	2	5	24	36	21	41	27
Care in therapy clinic for patient with special needs (per clinic)	**65**	–	–	–	2	–	–	7	4	11	47
Others	**9,390**	358	447	629	824	1,092	1,420	1,852	1,487	2,344	2,039

In the outpatient setting, the most common procedures performed on PD patients were physical therapy in patients with neurokinetic-functional disorders without systemic complications, with motor changes, and with neurokinetic-functional disorders with systemic complications. Physical therapy in patients with neurokinetic-functional disorders without systemic complications was performed on 17,616 patients (33.2%), with an average of 2,746 patients per year ( Table 3 ). When analyzing by years after the index date, it is notable that diagnostic procedures are most frequently performed in the first year, while physical therapy is conducted over a longer period ( Table 4 ).

**Table 4:** Procedures performed in the outpatient setting for patients with Parkinson's disease in the Brazilian Unified Health System by years after the index date

	Years after the index date								
	1-year	2-year	3-year	4-year	5-year	6-year	7-year	8-year	9-year
Physical therapy in patient with neurokinetic functional disorders without systemic complications	14,199	3,940	2,482	1,704	1,100	695	413	211	206
Physical therapy in patient with motor changes	13,035	3,115	1,992	1,371	905	550	330	148	126
Physical therapy in patient pre- and postsurgery with skeletal muscle dysfunction	9,536	1,881	1,175	724	438	284	199	73	67
Physical therapy in patient with neurokinetic unctional disorders with systemic complications	9,679	2,640	1,588	1,065	684	484	306	138	123
Patient intensive treatment in physical rehabilitation (1 patient-day shift; 20 calls -month)	4,231	1,471	1,083	756	511	342	198	109	105
Intensive care: follow-up of patient in physical rehabilitation (1 patient per day shift; 15 calls per month)	1,723	618	389	263	184	148	97	48	36
Consultation of higher-level professional in primary care (except medical)	82	19	4	10	4	1	–	1	–
Medical consultation on specialized attention	2,749	1,248	734	540	375	228	140	84	93
Computed tomography of skull	909	137	89	75	50	32	19	13	12
Magnetic Resonance of the skull	1,831	275	217	136	94	60	26	11	17
Care: monitoring on rehabilitation in multiple deficiencies	1,017	226	145	125	89	59	47	25	16
Physical therapy in patient pre- posteurosurgery	602	166	103	84	57	29	18	6	5
Teleconsultation by higher level professionals in specialized attention (except medical)	605	127	103	82	50	29	29	14	28
Household assistance by multi professional team in specialized attention	246	82	49	31	21	18	13	6	19
Individual therapy	256	95	67	58	45	32	20	17	17
Household assistance by multi professional team	152	54	33	15	11	1	1	1	2
Medical care in emergency care unit	84	18	12	9	12	7	3	4	2
Care in therapy clinic for patient with special needs (per clinic)	41	12	5	4	4	1	2	–	4
Others	6,908	1,565	1,151	789	536	373	235	123	176

#### *Treatment patterns*

Regarding treatment patterns, data from the APAC claims were available for 19,566 patients. The most used drugs for PD patients were pramipexole (8,921 patients; 45.6%), amantadine (5,096 patients; 26.0%), and entacapone (3,340 patients; 17.1%) ( Table 5 ).

**Table 5:** List of drugs used per patients with Parkinson's disease in the Brazilian Unified Health System

	N	(%)
Amantadine	5,096	26.0%
Bromocriptine	13	0.1%
Clozapine	83	0.4%
Entacapone	3,340	17.1%
Pramipexole	8,921	45.6%
Rasagiline	1,116	5.7%
Selegiline	972	5.0%
Tolcapone	12	0.1%
Trihexyphenidyl	13	0.1%
***Total available information***	**19,566**	**100.0%**

**Figure 2:**
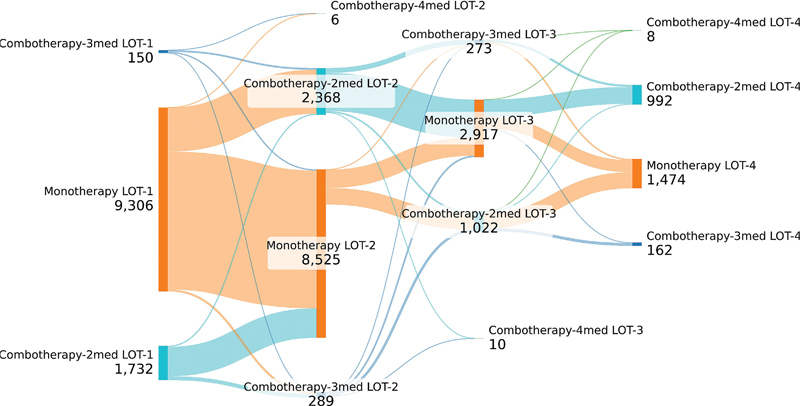
Note: *Only considering drugs prescribed under an Outpatient Procedure Authorization (Autorização de Procedimentos Ambulatoriais, APAC, in Portuguese) claim. Sankey diagram of PD treatment* in the Brazilian Unified Health System.

As this analysis is based solely on APAC claims, medications commonly used in the treatment of PD—such as levodopa, typically dispensed through the Basic Component of Pharmaceutical Assistance—were not captured in the dataset. Therefore, treatment patterns described here reflect only high-cost medications authorized through APAC. Among the PD patients included, most ( *N* = 9,306) initiated treatment with monotherapy, defined in this context as the use of a single high-cost drug (such as pramipexole, amantadine, or entacapone). Most of these patients remained on monotherapy in the second line of treatment. Notably, among those who transitioned to combination therapy in the second line, monotherapy was again the most frequent approach in the third line ( Figure 2 ).

For PD patients who received combination therapy as their initial treatment, the majority ( *N* = 1,732) were administered a regimen of 2 medications, while 150 were treated with a regimen of 3 drugs. Most patients treated with 2 medicines in line of therapy (LOT)-1 switched to monotherapy in the 2nd line, whereas most patients treated with 3 medications in LOT-1 continued with combination therapy in the 2nd line ( Figure 2 ).

## DISCUSSION

Parkinson's disease is a chronic, progressive neurodegenerative disorder that significantly affects patients and their families. It is the second most common neurodegenerative disease globally. 16 In Brazil, the absence of mandatory PD reporting complicates accurate prevalence estimates. 17 18

Age is a key factor in PD incidence, which increases with advancing age and typically affects individuals over 60. 19 In the current study, the mean age at the index date was of 65.4 ± 13.6 years, which is consistent with previous findings. A slight male predominance (53.0%) was observed, which is also supported by the literature. 19

Nearly half of the patients in the present study resided in the Southeast region of Brazil (48.8%), which may reflect more than just population distribution. This concentration likely also points to regional disparities in healthcare access, diagnostic infrastructure, and data reporting practices. The Southeast region, which includes major urban centers such as São Paulo and Rio de Janeiro, benefits from a higher density of specialized healthcare services and neurologists, as well as more robust health information systems. In contrast, the North and Midwest regions often face challenges such as limited availability of specialists, geographic barriers, and under-resourced healthcare facilities, which may contribute to underdiagnosis and underreporting of PD cases. These disparities have been well-documented in Brazilian health literature and underscore the urgent need for more equitable distribution of neurological care and diagnostic resources across the country. 20 21 22

More than half of the patients (55.5%) experienced multiple hospitalizations, with a mean of 5.1 ± 11.3 PD-related admissions. This notably high rate likely reflects not only the clinical complexity and progressive nature of PD but also systemic challenges within the healthcare system. Factors such as delayed access to outpatient care, lack of integrated care pathways, and regional disparities in service availability may contribute to avoidable hospital admissions and prolonged disease management. The mean hospital stay was of 12 ± 59 days, which further underscores the burden PD places on the public healthcare system. 23 The most frequent inpatient procedures were related to the medical management of PD symptoms, pharmacologic adjustments, and long-term neurological care, consistent with the disease's multifaceted progression. Hospitalizations are often needed due to acute complications such as infections, falls, and cognitive decline, which are common in advanced stages of PD. 24

In the outpatient setting, physical therapy was the most frequently recorded intervention, particularly for patients with progressive motor symptoms. This is consistent with clinical guidelines recommending regular physical therapy to improve motor function and reduce disability in PD. 25 26 Furthermore, the high demand for physical therapy in the treatment of PD patients, highlights the critical role of rehabilitation services in the management of this neurodegenerative condition. 27

A marked reduction in both inpatient (−30.1%) and outpatient (−25.8%) procedures was observed in 2020, likely due to the coronavirus disease 2019 (COVID-19) pandemic. This decline reflects the widespread disruption of healthcare services globally, driven by lockdown measures, reallocation of healthcare resources, and patient hesitancy to seek in-person care. In Brazil, these effects were particularly pronounced in the public health system, where elective procedures and routine follow-ups were postponed or canceled to prioritize COVID-19 response efforts. For individuals with PD such disruptions may have led to delays in rehabilitation, interruptions in medication management, and worsening of motor and non-motor symptoms. These consequences are especially concerning given the progressive nature of PD and the importance of continuous, multidisciplinary care in maintaining functional independence and quality of life. 28 29 30

Regarding pharmacological treatment, the current study analyzed only high-cost medications captured through APAC claims, which excludes drugs dispensed via the Basic Component of Pharmaceutical Assistance—most notably, levodopa. As levodopa is the cornerstone of PD treatment, 31 its absence from the dataset resulted in underreporting of its use and an overrepresentation of other therapies such as pramipexole, amantadine, and entacapone. These findings reflect prescribing patterns within the APAC system rather than the full spectrum of PD treatment in the public healthcare system.

Among the patients included in the study, most initiated therapy with a single high-cost drug. While this is referred to as “monotherapy” in the context of APAC data, it does not account for concurrent use of levodopa or other medications not captured in the dataset. Many patients with PD may remain on levodopa monotherapy for extended periods before requiring adjunctive therapy, 32 but this treatment trajectory could not be assessed in our analysis due to data limitations.

The progressive use of multiple high-cost medications over time likely reflects the natural course of PD and the increasing complexity required to manage its symptoms effectively. 33 However, this pattern should be interpreted cautiously, as it does not capture the full therapeutic landscape. Although specific procedural data on deep brain stimulation (DBS) were not available in this dataset, DBS is a key therapeutic option for patients with advanced PD who experience motor complications unresponsive to pharmacological treatment. 34 The significance of DBS in this context underscores the critical need to ensure equitable access to advanced interventions within the public healthcare system. Nevertheless, several barriers may hinder access to DBS, including the limited number of specialized centers, intricate evaluation protocols, high procedural costs, and geographic challenges—particularly for patients with severe mobility impairments. 35 36

The present study should be interpreted considering several limitations. As with most retrospective analyses, the data may be incomplete. Parkinson's disease cases were identified solely through ICD-10 code G20, given the non-mandatory reporting of PD in Brazil, which limits the ability to estimate prevalence accurately. To improve diagnostic specificity, only patients with at least two core procedures were included; however, this may have excluded individuals with recent diagnoses or limited healthcare utilization, potentially underrepresenting early-stage PD.

The analysis included only data from the public healthcare system, which may not capture the full range of healthcare resource utilization in Brazil. As a result, the findings are likely more representative of patients with severe disease who require inpatient or outpatient care. Furthermore, the database does not provide information on reasons for loss to follow-up, such as death or treatment discontinuation, nor does it include clinical variables like disease duration, severity, or Unified Parkinson's Disease Rating Scale (UPDRS) scores. This lack of clinical detail limits our ability to stratify patients by disease stage or monitor progression over time.

While dispensing records are available, they do not confirm adherence, though regular intervals of medication dispensation may serve as a proxy for continued use. Variability in drug availability due to supply chain issues, policy changes, and regional differences, as well as potential out-of-pocket purchases, further limit the completeness of medication data.

In addition, advanced therapies such as DBS may be underrepresented in the dataset, as these treatments are less frequently recorded in administrative claims or may be provided through alternative funding mechanisms not captured in the database.

In summary, the current study enhances the understanding of PD care in the public health system in Brazil, emphasizing the importance of effective and accessible management strategies to address the significant burden of the disease. Future research should concentrate on early interventions and continuous monitoring to reduce the frequency of hospitalizations and associated costs, as well as exploring the impact of regional and socioeconomic factors on the prevalence and treatment of the disease. Policymakers should prioritize expanding access to physiotherapy, ensuring equitable medication distribution, and supporting advanced interventions to improve outcomes for individuals living with PD.
